# Beyond Hematologic Malignancies: Colorectal Cancer as a Solid Tumor Manifestation of Inherited Bone Marrow Failure Syndromes

**DOI:** 10.3390/ijms262010105

**Published:** 2025-10-17

**Authors:** Sara Cagliano, Marta Potenza, Marta La Vecchia, Steven R. Ellis, Irma Dianzani, Anna Aspesi

**Affiliations:** 1 Department of Health Sciences, Università Del Piemonte Orientale, 28100 Novara, Italy; 20029319@studenti.uniupo.it (S.C.); 20023900@studenti.uniupo.it (M.P.); marta.lavecchia@uniupo.it (M.L.V.); irma.dianzani@med.uniupo.it (I.D.); 2 Department of Biochemistry and Molecular Genetics, University of Louisville, Louisville, KY 40292, USA; steven.ellis@louisville.edu

**Keywords:** inherited bone marrow failure syndromes, colorectal cancer, Fanconi anemia, Diamond-Blackfan anemia syndrome, dyskeratosis congenita, Shwachman-Diamond syndrome, cancer predisposition, DNA repair defects, ribosomal stress

## Abstract

Inherited Bone Marrow Failure Syndromes (IBMFS) encompass a group of rare genetic disorders characterized by intrinsic hematopoietic stem cell defects, leading to impaired hematopoiesis and increased predisposition to malignancies, particularly hematologic cancers. As advances in supportive care and hematopoietic stem cell transplantation have extended patient survival, there is growing recognition of an elevated risk of solid tumors, including colorectal cancer (CRC), within this population. Epidemiologic data, although limited by small cohort sizes, suggest the need for earlier and more intensive CRC surveillance protocols tailored to IBMFS patients, who tend to develop CRC at younger ages compared to the general population. Among IBMFS, the most robust association with CRC has been reported in Diamond–Blackfan anemia syndrome (DBAS) and Fanconi anemia (FA), while emerging evidence suggests a potential link in dyskeratosis congenita (DC) and Shwachman–Diamond syndrome (SDS). The pathophysiological basis involves defective DNA repair mechanisms, telomere dysfunction, ribosomal protein abnormalities, and impaired cellular stress responses, each contributing to genomic instability and malignant transformation. The understanding of the molecular mechanisms underpinning the association between IBMFS and CRC may provide a foundation for future targeted prevention and surveillance strategies and offer broader insights into colorectal carcinogenesis.

## 1. Introduction

Inherited bone marrow failure syndromes (IBMFS) constitute a heterogeneous group of rare genetic disorders characterized by defective hematopoiesis, varying degrees of bone marrow failure, and a markedly increased predisposition to malignancy [1,2,3,4]. These syndromes, including Diamond–Blackfan anemia syndrome (DBAS), Fanconi anemia (FA), dyskeratosis congenita (DC) and Shwachman–Diamond syndrome (SDS), collectively affect approximately 1 in 100,000 individuals worldwide [5,6,7]. While historically recognized primarily for their hematological manifestations and their elevated risk of hematologic malignancies, mounting evidence reveals a concerning pattern of increased solid tumor incidence (Table 1) that warrants both clinical attention and mechanistic understanding [8,9,10,11]. The cancer predisposition in IBMFS appears to be intrinsic to the fundamental cellular processes disrupted in these conditions, with ribosomal dysfunction, telomere maintenance defects, and DNA repair pathway abnormalities each contributing distinct oncogenic vulnerabilities. In DBAS, haploinsufficiency of ribosomal protein (RP) genes leads to ribosomal stress and aberrant p53 activation, creating a cellular environment that paradoxically promotes both growth inhibition and oncogenic transformation [12]. Conversely, the genomic instability observed in Fanconi anemia and dyskeratosis congenita arises from defects in DNA repair and telomere maintenance, respectively, driving carcinogenesis through accumulated genetic damage [13,14].

This broad solid tumor susceptibility has transformed the clinical management paradigm for IBMFS patients, necessitating comprehensive cancer surveillance protocols that extend throughout the patient’s lifetime and encompass multiple organ systems. Importantly, the cancer predisposition associated with germline pathogenic variants in FA genes is not limited to individuals with classic biallelic mutations manifesting as Fanconi anemia. Heterozygous carriers of pathogenic variants in FA pathway genes—most notably *FANCD1/BRCA2* and *FANCN/PALB2*—are at increased risk for various cancers, particularly breast, ovarian, and pancreatic malignancies [15,35]. Although the link with CRC remains less definitive, some studies suggest a potential association [36,37,38]. Emerging evidence also points to additional FA genes, such as *RAD51D*, *FANCM*, and *FANCJ/BRIP1*, as contributors to cancer risk in the heterozygous state [15,39]. The underlying mechanism likely involves haploinsufficiency or partial disruption of DNA interstrand crosslink (ICL) repair, leading to genomic instability even in the absence of overt marrow failure.

Among the spectrum of solid tumors associated with IBMFS, colorectal cancer (CRC) has emerged as a particularly significant clinical concern, especially in patients with DBAS. Patients with DBAS have a 4.8-fold increased relative risk of developing cancer compared to the general population, with a striking 45-fold increased relative risk for CRC [28]. This association is particularly remarkable given that CRC typically manifests in IBMFS patients at ages decades younger than the general population, often occurring in the third and fourth decades of life when standard screening protocols are not routinely implemented [27,40].

Colorectal cancer is the third most commonly diagnosed cancer worldwide and the second leading cause of cancer-related death [41]. As CRC incidence rises worldwide and survival of IBMFS patients improves, understanding cancer risk in this population becomes increasingly relevant.

The heightened susceptibility to CRC in IBMFS patients carries important and wide-ranging clinical consequences. First, the early onset of CRC in IBMFS patients necessitates a reevaluation of existing surveillance protocols, as standard age-based screening guidelines are inadequate for identifying high-risk individuals within this population. Second, elucidating the molecular pathways driving CRC in IBMFS may yield novel insights into the mechanisms of colorectal carcinogenesis more broadly, potentially uncovering new biomarkers or therapeutic targets [42]. Third, the treatment of CRC in IBMFS patients poses unique challenges, given their underlying hematologic fragility and potential hypersensitivity to DNA-damaging agents, which complicates the use of conventional chemotherapeutic regimens.

Despite growing recognition of the association between IBMFS and CRC, substantial knowledge gaps remain, particularly concerning the precise molecular mechanisms that connect bone marrow failure to colorectal carcinogenesis. As our understanding of IBMFS continues to evolve from isolated hematologic disorders to complex cancer predisposition syndromes, the development of comprehensive, multidisciplinary care strategies becomes essential to optimize outcomes for these patients throughout their lifespans. This narrative review synthesizes current evidence regarding the link between IBMFS and CRC risk, examining the epidemiological data, molecular mechanisms, and clinical implications for patient care. Only articles in English published between 1995 and July 2025 available on PubMed were considered. The literature search included keywords such as inherited bone marrow failure syndromes, Fanconi anemia, Diamond–Blackfan anemia, dyskeratosis congenita, and Shwachman–Diamond syndrome, alone or in combination with colorectal cancer. By integrating insights from registry observations, functional studies, and clinical research, we discuss the emerging understanding of how fundamental cellular processes disrupted in IBMFS contribute to colorectal carcinogenesis.

## 2. Overview of IBMFS

The four major IBMFS—FA, DC, DBAS and SDS—collectively account for approximately 30% of pediatric bone marrow failure cases [43]. With advancements in genetic testing and improved survival rates of affected individuals into adulthood, IBMFS are now increasingly recognized in adult populations. However, delayed diagnosis in adults remains a significant clinical challenge, often due to the heterogeneous presentations, subtle phenotypic features, and overlapping symptoms with acquired bone marrow disorders [44].

The National Cancer Institute (NCI) Inherited Bone Marrow Failure Syndromes (IBMFS) cohort study, initiated in 2002, is the largest prospective study of its kind, enrolling patients with FA, DC, DBAS, and SDS under a unified, comprehensive protocol. After fifteen years of follow-up, this cohort has provided crucial insights into cancer incidence patterns across IBMFS and has documented that patients with these syndromes have high risks of cancer development, with distinct patterns of malignancy for each syndrome [17]. Additional important registries include the Diamond–Blackfan Anemia Registry of North America, the International Fanconi Anemia Registry, and various national registries that have contributed to our understanding of cancer risks in IBMFS [21,27,45]. These registries have been instrumental in identifying CRC as an emerging cancer risk across multiple IBMFS, and in highlighting the need for specific cancer surveillance recommendations.

### 2.1. Fanconi Anemia

Fanconi anemia is caused by germline biallelic mutations in one of the 22 currently recognized FANC genes (*FANCA-W*), which encode proteins involved in the DNA ICL repair pathway [46,47]. Of these, mutations in 20 genes follow an autosomal recessive inheritance pattern, whereas *FANCB* is X-linked and *FANCR/RAD51* may harbor a heterozygous variant that exerts a dominant-negative effect [48,49]. The most commonly affected gene is *FANCA*, accounting for approximately 60–70% of cases [50]. The FA pathway is essential for maintaining genomic stability by repairing DNA ICLs through homologous recombination. Defects in this pathway lead to chromosomal instability, hypersensitivity to DNA crosslinking agents, and characteristic chromosomal breakage patterns when cells are exposed to mitomycin C or diepoxybutane [51]. Patients with FA have a dramatically increased cancer risk, with a cumulative incidence of 20–30% by age 40 years [21,52]. The malignancy spectrum includes acute myeloid leukemia (AML), myelodysplastic syndrome (MDS), and solid tumors, particularly squamous cell carcinomas of the head and neck, gynecologic tract, and gastrointestinal system [16,17,53,54]. Solid tumors typically occur later in life than hematological features, with head and neck squamous cell carcinoma (HNSCC) being the most frequently reported, showing a 500-fold increased risk [21,55,56]. Hepatocellular carcinoma and breast cancer are also reported with increased frequency [15,57]. Notably, biallelic mutations in certain FA pathway genes, such as *FANCS/BRCA1*, *FANCO/RAD51C*, *FANCR/RAD51*, and *FANCM* can cause an FA-like disorder that shares the cancer predisposition of classical FA and, in some cases, the associated developmental abnormalities, but lacks bone marrow failure [58]. *FANCM* is not essential for the formation or stabilization of the FA core complex, and both monoallelic and biallelic loss-of-function mutations have been linked to an increased risk of breast cancer, with biallelic mutations likely conferring a stronger effect [59,60].

While historically underrecognized, CRC has emerged as a significant solid tumor risk in FA patients. Large registry studies have documented CRC cases in FA cohorts, although precise incidence rates remain difficult to establish due to the rarity of the syndrome and competing mortality from bone marrow failure and other malignancies. Case reports describe CRC development in FA patients as early as the third and fourth decades of life, representing a substantially younger age of onset compared to sporadic CRC [61]. The defective DNA repair mechanisms characteristic of FA likely contribute to the increased CRC susceptibility, as ICL repair defects can lead to accumulation of mutations in colorectal epithelium.

Of particular relevance to CRC risk assessment is the emerging understanding of cancer susceptibility in heterozygous carriers of FA pathogenic variants. While most heterozygous carriers do not appear to have significantly increased cancer risks [62,63], susceptibility to cancer, including CRC, is well established for carriers of heterozygous pathogenic variants in *FANCS/BRCA1*, *FANCD1/BRCA2*, *FANCN/PALB2*, *FANCJ/BRIP1,* and *FANCO/RAD51C* [62,64]. Additional FA pathway genes, including *FANCE* and *FANCM*, have been implicated in familial CRC cohorts [61,65,66].

### 2.2. Dyskeratosis Congenita

Dyskeratosis congenita results from mutations in genes that encode components of the telomerase complex or telomere-associated proteins essential for telomere length maintenance [67]. The most commonly affected genes include *DKC1* and *POLA1* (X-linked), *TERC*, *TERT*, and *TINF2* (autosomal dominant), and *NOP10*, *NHP2*, and *WRAP53* (autosomal recessive) [23,24]. These mutations lead to accelerated telomere shortening, premature cellular senescence, and stem cell dysfunction. The resulting genomic instability and impaired cellular proliferation contribute to both the bone marrow failure phenotype and cancer predisposition [68,69]. Dyskeratosis congenita manifests with a classic triad of mucocutaneous features—reticulate skin pigmentation, nail dystrophy, and oral leukoplakia—but these features may be subtle or absent. The systemic nature of the disease reflects the impact of telomere dysfunction on high-turnover tissues. Bone marrow failure is the most common life-threatening complication, while other organ systems, including the lungs, liver, and gastrointestinal tract, may also be affected [70].

Patients with DC have a high propensity for developing malignancies, that occur in approximately 40–50% of patients [25,71]. The cancer spectrum includes both hematologic malignancies (AML, MDS, Hodgkin lymphoma) and solid tumors. Solid tumors characteristically include squamous cell carcinomas of the head and neck, lung, cervix, and anus [16,17,72]. Colorectal cancer has been reported in DC patients, though data remain limited due to the syndrome’s rarity. These cancers are often aggressive and difficult to treat due to the underlying tissue fragility and impaired tissue healing associated with DC [73]. One illustrative case involved a male patient with a confirmed *DKC1* mutation who developed rectal adenocarcinomas at ages 16 and 18, without a family history of CRC [74]. This case highlights not only the early onset and aggressive nature of CRC in DC but also the importance of surveillance in at-risk individuals.

### 2.3. Diamond–Blackfan Anemia Syndrome

Diamond–Blackfan anemia syndrome (previously Diamond–Blackfan anemia, DBA) is a rare IBMFS with an estimated incidence of 5 to 7 cases per million live births [6,75]. Clinically, DBAS presents most often in infancy with macrocytic anemia, and is frequently associated with congenital anomalies (such as craniofacial, upper limb, and cardiac defects) and with an elevated lifetime risk of malignancies, most notably MDS and AML [76]. The pathophysiology of DBAS is fundamentally rooted in defective ribosome biogenesis, establishing it as the prototypical ribosomopathy. The disorder results from heterozygous mutations in genes encoding RPs, affecting both large and small ribosomal subunit components. The most commonly implicated genes include *RPS19* (25% of cases), *RPL5*, *RPL11*, *RPL35A*, *RPS10*, *RPS17*, *RPS24*, and *RPS26*, among others [26]. These mutations lead to RP haploinsufficiency, which disrupts the normal assembly and function of ribosomes [26,77]. The molecular cascade begins with impaired ribosome biogenesis, which triggers nucleolar stress and activates the p53 pathway [78]. This activation results in enhanced apoptosis specifically in erythroid progenitor cells, leading to the characteristic erythroid failure observed in DBAS [79]. The tissue-specific manifestation of this ribosomal defect in erythropoiesis, despite ribosomes being essential for protein synthesis in all cells, remains an area of active investigation. Current hypotheses suggest that erythroid precursors may be particularly vulnerable to ribosomal stress due to their high proliferation rate and intensive protein synthesis requirements during hemoglobin production [80,81].

Registry data demonstrate that patients with DBAS have a 4.8-fold higher relative risk of developing malignancies compared to the general population, with an overall cumulative cancer incidence of 13.7% by age 45 years [9]. The two most prevalent solid tumors in DBAS patients are CRC and osteogenic sarcoma [82]. The Diamond–Blackfan Anemia Registry of North America (DBAR), the largest established DBA cohort with prospective follow-up since 1991, has provided the most comprehensive data on CRC incidence in DBA. Registry data demonstrate that CRC represents a significant and early-onset malignancy risk, with a median age of onset of 41 years (range 20–51 years) [28]. This early onset has prompted the development of preliminary CRC screening recommendations for DBAS patients, including consideration of colonoscopic screening beginning at age 20 years [6]. For patients with normal findings on initial colonoscopy, a follow-up interval of every five years is advised. In cases where abnormalities are detected, follow-up should adhere to established colorectal screening guidelines [83]. In patients who have undergone hematopoietic stem cell transplantation (HSCT) prior to age 20, it has been recommended that the first screening colonoscopy be performed within one year of HSCT, if clinically feasible, or prior to age 20 [6,28].

Among RP genes associated with DBAS, *RPS20* represents a notable exception as germline heterozygous mutations in this gene are not typically associated with hematologic manifestations but rather confer a predisposition to cancer, particularly to hereditary nonpolyposis colorectal carcinoma without mismatch-repair deficiency. A truncating germline mutation (c.147dupA p.Val50Serfs*23) in *RPS20* was identified in a four-generation Finnish pedigree with multiple cases of CRC [84]. No tumor showed loss of the wild-type allele, indicating that heterozygosity was sufficient to increase CRC risk. The mutation caused a defect in pre-ribosomal RNA maturation, as expected for DBAS genes [84]. Subsequent case series and screening studies identified additional *RPS20* null and pathogenic missense mutations in early-onset CRC patients, but not in controls [85,86,87,88]. Interestingly, two missense variants affecting codon 84, both with a deleterious effect on protein function, have been reported in two DBAS patients who presented with transfusion-dependent anemia during the first month of life [89].

### 2.4. Shwachman–Diamond Syndrome

Shwachman–Diamond syndrome is a rare autosomal recessive disorder primarily characterized by exocrine pancreatic insufficiency, neutropenia, and skeletal abnormalities [90]. Approximately 90% of patients with SDS harbor biallelic mutations in the *SBDS* gene, which encodes a protein involved in ribosome biogenesis and mitotic spindle stabilization. *SBDS* mutations lead to defective ribosome maturation, impaired protein synthesis, and cellular dysfunction, placing SDS among the group of genetic diseases known as ribosomopathies. Additional genes, including *DNAJC21*, *SRP54*, and *EFL1*, have been identified in SBDS-negative cases [30]. Under normal conditions, SBDS, in cooperation with the GTPase EFL1, catalyzes the release of eIF6, which otherwise blocks the joining of 40S and 60S subunits. When SBDS is deficient or dysfunctional, eIF6 remains bound to the 60S subunit, preventing proper ribosome assembly and stalling translation initiation. This defect leads to inefficient ribosome formation and a global reduction in translation, resulting in hematopoietic failure [91,92].

Patients with SDS demonstrate a substantially increased predisposition to myeloid malignancies, particularly MDS or AML, with a cumulative lifetime risk probably exceeding 30% [93,94,95]. Data on gastrointestinal malignancies and other solid tumor risks have been less well-characterized due to the syndrome’s rarity and patient survival patterns [17,32,33,34,96].

Recent evidence suggests that germline pathogenic variants in *SBDS* may contribute to solid tumor development also in the heterozygous state. A large-scale study of 17,904 Chinese lung cancer patients identified *SBDS* as the most frequently mutated gene among cancer-susceptibility genes, with a prevalence of 1.37% [97]. The *SBDS* c.184A>T (p.Lys62*) pathogenic variant demonstrated a significant association with increased lung cancer risk, showing an odds ratio of 4.8 in case–control analysis compared to the gnomAD East Asian population [97]. The same germline variant was reported in one patients with malignant pleural mesothelioma [98] and in a pediatric patient with Ewing sarcoma [99]. These findings raise the possibility that heterozygous *SBDS* pathogenic variants may be predisposed to a broader range of solid tumors. In this context, preliminary data from our cohort of 292 patients with early-stage colorectal tumors identified two cases of heterozygous pathogenic variants in *SBDS* (manuscript in preparation). These findings indicate that heterozygous *SBDS* variants may contribute to the genetic predisposition to solid tumors, including CRC.

## 3. Molecular Mechanisms Underlying Colorectal Carcinogenesis in IBMFS

**Figure 1:**
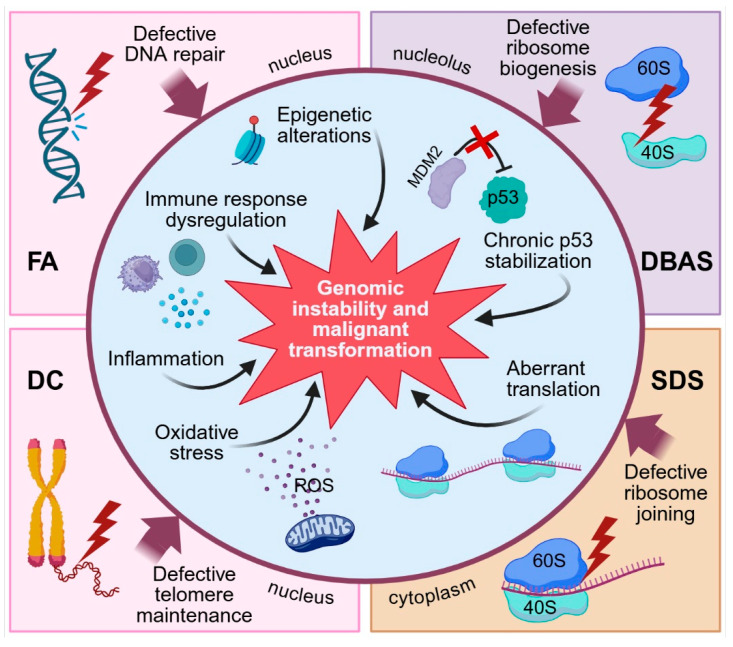
Molecular mechanisms underlying IBMFS pathophysiology and CRC risk (created with BioRender.com).

The pathophysiological mechanisms underpinning the increased CRC risk in IBMFS involve complex interactions between defective cellular pathways, genomic instability, and disrupted homeostatic processes. Each syndrome exhibits distinct molecular abnormalities that may contribute to colorectal carcinogenesis through overlapping yet syndrome-specific mechanisms (Figure 1).

### 3.1. DNA Repair Defects and Genomic Instability

Fanconi anemia represents a paradigmatic example of how inherited defects in DNA repair machinery can predispose individuals to cancer development through genomic instability. The FA pathway, comprising at least 22 complementation groups (FANCA-FANCW), constitutes a specialized DNA repair network that maintains genomic integrity primarily through the resolution of DNA ICLs [100]. The FA pathway is activated through a well-coordinated sequence of molecular events, initiated by the recognition of DNA ICLs during replication. The FA core complex, a multisubunit E3 ubiquitin ligase, is composed of FANCA, FANCB, FANCC, FANCE, FANCF, FANCG, and FANCL [101]. The catalytic activity resides in the FANCL subunit, which, in cooperation with the E2 ubiquitin-conjugating enzyme UBE2T, mediates the monoubiquitination of the FANCD2–FANCI (ID2) complex. This monoubiquitination event is a pivotal step in activating the DNA damage response to ICLs [102]. The monoubiquitinated ID2 complex serves as a platform for the recruitment of downstream effector proteins, including nucleases (e.g., FANCP/SLX4 and FANCQ/ERCC4), helicases (e.g., FANCJ/BRIP1 and FANCM), and homologous recombination factors (e.g., FANCD1/BRCA2, FANCN/PALB2, and FANCO/RAD51C) [102,103,104].

Recent genomic analyses have revealed that FA pathway deficiency creates a distinctive mutational signature characterized by increased numbers of structural variants, particularly small deletions [13]. This signature reflects the faulty response to unrepaired ICLs, which can lead to replication fork collapse and formation of DNA double-strand breaks. In FA cells these lesions are repaired through error-prone repair mechanisms, such as non-homologous end joining and microhomology-mediated end joining, which contribute to genomic instability [105,106]. Dysfunctions in the FA pathway compromise the cellular response to endogenous DNA damaging agents, particularly reactive aldehydes produced through normal metabolic processes. For example, acetaldehyde—generated during alcohol metabolism—can spontaneously react with DNA to form ICLs [107].

At the molecular level, deficiency in FA pathway components in colorectal epithelium creates a permissive environment for DNA damage that drives carcinogenesis. The continuous exposure to dietary and metabolic aldehydes, combined with the inability to repair resulting ICLs, leads to the accumulation of mutations in critical tumor suppressor genes and oncogenes [108]. Additionally, the chromosomal instability associated with FA may promote aneuploidy and loss of heterozygosity events that accelerate the progression from adenoma to carcinoma [109].

Dyskeratosis congenita is characterized by genomic instability arising from defective telomere maintenance rather than classical DNA repair pathways [70]. Critically shortened telomeres lose their protective function and are misrecognized by the DNA damage response machinery as double-strand breaks. This triggers inappropriate end-to-end chromosome fusions, resulting in dicentric chromosomes that undergo breakage during mitosis, creating a cycle of genomic rearrangements [110]. The accumulation of these chromosomal aberrations provides the foundation for neoplastic transformation. This mechanism explains the paradoxical observation that both very short and very long telomeres can be associated with cancer, depending on the cellular context and the ability to bypass senescence checkpoints [71].

Single-cell RNA sequencing studies in DBA cells indicate that the imbalance between rapid DNA replication and insufficient protein synthesis due to RP haploinsufficiency might trigger replication stress, leading to DNA damage, as reflected by anti-γH2AX positivity, and elevated pro-apoptotic gene expression [111].

In SDS, the deficiency in the *SBDS* gene leads to destabilization of the mitotic spindle, which can result in aberrant cell division processes, such as aneuploidy, where cells have an abnormal number of chromosomes, and centrosomal amplification, leading to multipolar spindles [112,113]. These events can promote the accumulation of further genetic damage over time. The connection between genomic instability and cancer in SDS is further evidenced by the observation of clonal cytogenetic abnormalities in the bone marrow of affected patients [114]. Leukemias that arise in individuals with SDS often exhibit aneuploidy and complex chromosomal aberrations [115]. The accumulation of such genetic alterations can lead to the loss of critical cellular checkpoints, such as the p53 tumor suppressor pathway, thereby promoting uncontrolled cell proliferation and tumorigenesis [93].

### 3.2. p53 Pathway Activation and Cellular Stress Response

Although DC, FA, DBAS, and SDS originate from disruptions in separate cellular processes, i.e., telomere biology, DNA repair, ribosome biogenesis and function, respectively, they converge on a shared pathogenic mechanism: chronic activation of the p53 stress response [116]. Increase in p53 level drives bone marrow failure by impairing self-renewal and promoting apoptosis or senescence of hematopoietic stem and progenitor cells (HSPCs) [79,117,118,119,120]. Theoretically, p53 activation should protect cells against malignant transformation by inducing cell cycle arrest or apoptosis. However, the selective pressure exerted by chronic p53 activation in IBMFS patients may favor the emergence of cells with p53 mutations or cells that have developed mechanisms to bypass p53-mediated growth arrest [121,122].

Cells with FA pathway deficiencies exhibit constitutive p53 activation due to their fundamental inability to properly repair DNA ICLs and other DNA lesions. The defective FA DNA repair pathway leads to accumulation of DNA damage that continuously triggers ATM-mediated p53 phosphorylation [123,124]. This persistent genotoxic stress results in elevated levels of p53 and its downstream effectors, and in increased production of reactive oxygen species (ROS), which create a feed-forward loop that further amplifies p53 signaling [125]. Importantly, FA cells demonstrate hypersensitivity to DNA crosslinking agents, which enhances p53-mediated apoptotic responses.

In DC, defective telomere maintenance leads to critically short telomeres that trigger persistent DNA damage responses. The shortest telomeres are recognized as double-strand breaks, activating ATM and ATR kinases that stabilize p53 [126]. This chronic telomere-induced DNA damage signaling results in sustained p53 activation, particularly pronounced in highly proliferative tissues such as the hematopoietic system [119]. Studies have demonstrated elevated p53 protein levels and increased expression of p53 target genes in DC patient cells [127,128].

Diamond–Blackfan Anemia Syndrome is characterized by RP haploinsufficiency that leads to the accumulation of unprocessed rRNA and impaired ribosomal subunit assembly within the nucleolus, activating a nucleolar stress response [129]. A key consequence is the stabilization and activation of p53, mediated by the sequestration of MDM2 by free RPs such as RPL5 and RPL11 [130]. Moreover, it has been recently reported that, under ribosomal or nucleolar stress, RPL22 promotes skipping of exon 6 in the MDM4 transcript by binding specific stem-loop RNA elements within intron 6. This exon skipping reduces full-length MDM4, thereby enhancing p53 activity and limiting proliferation [131,132]. DBAS patient cells show elevated p53 protein levels, increased p21 expression, and enhanced sensitivity to additional nucleolar stressors [133,134]. Loss or mutation of RPL22, which is rather frequent in CRC, interferes with the MDM4–p53 axis and leads to increased full-length MDM4, suppression of p53, and enhanced cell growth and drug resistance [131,135,136,137].

In SDS, dysfunction of the SBDS protein impairs ribosome maturation and leads to RPL5- and RPL11-mediated p53 stabilization similar to DBAS [138]. SDS cells demonstrate elevated p53 levels and increased expression of p53 target genes, particularly those involved in cell cycle control and apoptosis [138,139,140]. Furthermore, SDS cells show increased susceptibility to oxidative stress [141,142], which can further enhance p53 activation.

The constitutive stabilization and activation of p53 in IBMFS enforces cell cycle arrest, promotes apoptosis, and restricts cellular growth, thereby imposing a strong constraint on cellular proliferation, particularly within hematopoietic progenitors and other highly proliferative tissues. Under this pressure, cells that acquire somatic *TP53* mutations, or otherwise lose p53 activity, are able to bypass the p53-mediated checkpoints and gain a competitive growth advantage. For instance, the p53-Arg273His pathogenic variant exhibits dominant-negative properties inhibiting wild-type p53 through oligomerization and confers a gain-of-function phenotype that promotes carcinogenesis. In CRC this mutant p53 has been shown to regulate specific long non-coding RNAs and transcriptional programs that sustain self-renewal, invasion, and therapeutic resistance [143,144,145]. Selection and expansion of cells harboring *TP53* mutations is known to be responsible for clonal hematopoiesis and leukemic progression in IBMFS [146,147], and may also occur in non-hematopoietic tissues, including the intestinal epithelium.

### 3.3. Immune Dysfunction and Inflammation

Most IBMFS are characterized by constitutive activation of inflammatory pathways that can mediate stem cell exhaustion and clonal selection pressure. The pro-inflammatory microenvironment promotes genomic instability and suppresses normal hematopoiesis. Immune surveillance can also be impaired, facilitating immune evasion and malignant transformation. While there is no complete consensus on cytokine profiles, and studies report variable findings regarding inflammatory markers and interleukins, pro-inflammatory cytokines are generally increased across IBMFS subtypes [148,149] and may play a role in enhancing oxidative stress and DNA damage in IBMFS pathogenesis and carcinogenesis. The pro-inflammatory cytokine milieu associated with chronic p53 activation can promote a pro-tumorigenic microenvironment in the gastrointestinal tract.

In FA, abnormal production of pro-inflammatory cytokines has been observed in vitro, in vivo, and in patients [150,151]. Activation of TNF-α signaling represents the dominant inflammatory pathway. TNF-α is overexpressed due to dysregulated stress-response pathways such as NF-κB and MAPK [125]. Additionally, elevated levels of TGF-β3 suggest a hyperactivated TGF-β signaling pathway in the bone marrow microenvironment [152,153]. Both adults and children with FA show decreased B- and NK-cells compared to healthy individuals, and lower IgM [154,155].

For DC patients, the most consistently reported immunological abnormality in the literature is a marked reduction in B-cell and NK-cell counts, whereas T-cell lymphopenia is less common [154]. Dysgammaglobulinemia, characterized by decreased levels of IgG, IgM, or IgA, has been observed in some studies [156,157]. Elevated serum levels of G-CSF and Flt3L, along with reduced RANTES, have been linked to severe marrow failure in DC [154].

Unlike other IBMFS, DBAS has not shown significant changes in pro-inflammatory cytokines like TNF-α and IFN-γ in patients [154]. An earlier study reported no significant immunodeficiency in patients with DBAS [154]. However, subsequent research involving 128 children and adults with DBAS demonstrated reduced numbers of B-cells and NK-cells, along with decreased IgM levels; these findings were largely independent of steroid therapy [158]. Another study focusing on pediatric DBAS patients further confirmed low immunoglobulin levels in a subset of individuals [159].

Immune function in SDS patients is not severely affected [154], but neutrophils from SDS patients are reduced in number and have significantly impaired migratory activity [160]. SDS patients can exhibit elevated plasma levels of TGF-β, and upregulation of the TGF-β pathway has been observed [161], as well as elevated plasma levels of several pro-inflammatory cytokines involved in the mTOR/STAT3 pathway, such as IL-6, IL-8, and IL-12 [162].

### 3.4. Oxidative Stress

Oxidative stress represents another critical component of the cellular stress response in IBMFS. Reactive oxygen species are natural byproducts of mitochondrial energy metabolism and play a role in normal cellular signaling. However, excessive ROS can damage DNA, RNA, proteins, and cellular structures, contributing to genomic instability and cancer development. In FA, pathogenic variants in genes like *FANCA*, *FANCC*, and *FANCD2* are associated with increased ROS production, impaired mitochondrial function, and diminished ATP generation [125]. Cells deficient in the FA pathway show elevated oxidative DNA damage, chromosomal breakage, and apoptosis [163]. The FA pathway plays a critical role in regulating oxidative stress, and its inactivation impairs systemic antioxidant defenses and mitochondrial ROS-scavenging capacity. Loss of mitochondrial integrity adds to redox imbalance. Furthermore, pro-inflammatory cytokines released by FA cells amplify ROS production both in vitro and in vivo [164].

Telomere dysfunction in DC leads to mitochondrial abnormalities and impaired antioxidant defenses, resulting in accumulation of oxidative damage [165]. Lymphocytes isolated from DC patients show elevated levels of ROS, DNA damage response and apoptotic markers, especially after exposure to cytotoxic agents [127]. Experimental models have shown that oxidative stress exacerbates telomere attrition, contributing to genomic instability and cellular senescence in DC [127,166].

Oxidative stress has also been implicated in DBAS pathophysiology. Mutations that impair ribosomal biogenesis and function cause defective synthesis of globin chains in erythroid progenitors, leading to a marked decrease in globin production. Since heme synthesis remains unaffected, there is an imbalance between globins and heme within the cell and the excess free heme contributes to oxidative stress and apoptosis or ferroptosis of erythroid progenitors, thereby exacerbating the anemia observed in DBAS [80,167]. Cells with RP deficiency show a downregulation in the expression of genes involved in the protection against oxidative stress [168]. Studies using Rp-deficient murine and patient-derived cells revealed that elevated ROS production exceeds antioxidant capacity, leading to oxidative DNA damage, cellular senescence, and activation of DNA damage response pathways [169,170].

Impaired protein synthesis in SDS cells results in decreased expression of antioxidant enzymes including superoxide dismutase (SOD), catalase, and glutathione peroxidase, creating an oxidative imbalance favoring ROS accumulation [139]. A reduction in mitochondrial SOD2 levels has also been reported in a yeast model of SDS [171].

Although increased oxidative stress has been extensively documented in hematopoietic cells and blood of patients with IBMFS [80,125,127,169,172], there is currently no direct evidence that ROS levels are elevated in the intestinal epithelium of these individuals. Nevertheless, given the known role of ROS in colorectal carcinogenesis [173] and the systemic oxidative imbalance observed in IBMFS, it is plausible that oxidative damage could also occur in the colonic mucosa, thereby contributing to cancer risk.

### 3.5. Other Mechanisms Possibly Involved in Colorectal Carcinogenesis

Alterations in the translation of specific mRNAs have been implicated in DBAS, SDS and DC pathophysiology. Reduction in ribosome levels profoundly impairs translation of a specific subset of transcripts [174]. Downregulation of Rps19 or Rpl11 in murine erythroblasts caused deregulated translation initiation of specific transcripts, and this result was confirmed in erythroblasts cultured from DBA patients [175]. RPS19 knockdown in erythroid cells reduced GATA1 protein expression without significantly affecting total cellular protein levels. Notably, total *GATA1* mRNA levels were unchanged, consistent with the hypothesis that transcripts with complex 5′ UTR are inefficiently translated under conditions of limited ribosome abundance [176].

In SDS, the loss of SBDS function causes inefficient translation re-initiation, a mechanism that allows ribosomes to resume translation at a downstream start codon, of the mRNAs of the transcription factors C/EBPα and -β [177]. As a consequence, some protein isoforms of these transcripts are produced at reduced levels, leading to decreased MYC expression and impaired cellular proliferation that possibly contributes to the hematological phenotype of SDS [177].

A seminal study by Yoon et al. demonstrated that cap-dependent translation was not affected in a mouse model of DC, whereas Internal Ribosome Entry Site (IRES)-dependent translation was selectively impaired, reducing synthesis of mRNAs encoding key regulators such as p27(Kip1), Bcl-xL, and XIAP [178]. The same translational defects was observed in cells derived from DC patients [178]. Deficient translation of tumor suppressors and anti-apoptotic factors may create a permissive environment for genomic instability and uncontrolled proliferation that may drive oncogenesis [179].

Moreover, ribosomes purified from a cellular model of DC obtained by DKC1 knockdown, exhibited impaired translational fidelity [180], indicating an increased tendency to misincorporate amino acids or misread mRNAs during protein synthesis. This defect may contribute to the elevated cancer risk observed in DC.

Epigenetic dysregulation might be another contributor to the pathophysiology of IBMFS. FA cells exhibit significant alterations in their epigenetic regulatory machinery, characterized by marked downregulation of key DNA methyltransferases. Expression studies in peripheral blood mononuclear cells and bone marrow from FA patients show reduced levels of DNMT1, which maintains DNA methylation during replication, and DNMT3B, a tumor suppressor involved in establishing genomic methylation, compared to healthy controls [181]. The downregulation of epigenetic modifiers results in global DNA hypomethylation, particularly affecting tumor suppressor gene loci [181]. Interestingly, it has been proposed that vitamin D supplementation may offer a therapeutic strategy to slow disease progression and reduce cancer risk in FA patients, thanks to its ability to modulate immune responses and regulate the expression of genes like *FANCE* through epigenetic mechanisms [182].

## 4. Clinical Implications

The heightened CRC risk in IBMFS patients strongly support the need for tailored surveillance protocols, such as initiating colonoscopic screening well before standard age [6,28], and for management strategies based on precision oncology.

Treatment of CRC in IBMFS patients poses unique challenges. Patients with FA, in particular, exhibit severe hypersensitivity to DNA-damaging agents (e.g., alkylating chemotherapy, radiation), making conventional therapies highly toxic and often contraindicated. Surgery remains the primary curative modality for solid tumors in this population.

Poly (ADP-ribose) polymerase (PARP) inhibitors represent a promising class of therapeutic agents for CRC [183,184]. These inhibitors, originally developed for tumors with homologous recombination deficiencies such as BRCA-mutated breast and ovarian cancers, act by inducing synthetic lethality in cells with defects in the DNA damage repair (DDR) pathway. Such DDR alterations are frequently observed in CRC. Ongoing research is focused on defining the molecular subgroups of patients that are most likely to benefit from these therapies [183]. The use of PARP inhibitors in patients with FA raises serious concerns due to systemic toxicity. Since FA is caused by biallelic mutations in genes involved in homologous recombination (HR)-mediated DNA repair, all somatic cells are hypersensitive to DNA-damaging agents, including PARP inhibitors. As PARP inhibitors exploit HR deficiencies in tumors [184,185], their use in FA patients would likely result in widespread cytotoxic effects, making them clinically inappropriate in this context. In contrast, individuals who carry heterozygous germline mutations in FA genes without clinical FA, and who develop CRC with somatic loss of the wild-type allele, may represent a distinct subgroup in which PARP inhibitors could be therapeutically beneficial. In these patients, the tumor may exhibit synthetic lethality in response to PARP inhibition, while normal tissues retain sufficient FA pathway activity to tolerate treatment. This distinction underscores the importance of carefully differentiating between biallelic FA patients and heterozygous FA mutation carriers when considering therapies for CRC.

## 5. Conclusions

Inherited Bone Marrow Failure Syndromes are associated with a significantly increased risk of CRC, often manifesting at younger ages than in the general population. Future research priorities include prospective studies to better define CRC incidence and risk factors, establishment of surveillance protocols and development of personalized prevention and treatment strategies for optimal patient care.

## Figures and Tables

**Table 1:** Tumor types described in patients with IBMFS.

Disease	Affected Genes	Associated Solid Tumors
Fanconi Anemia	*FANCA*, *FANCB*, *FANCC*, *FANCD1*, *FANCD2*, *FANCE*, *FANCF*, *FANCG*, *FANCI*, *FANCJ*, *FANCL*, *FANCM*, *FANCN*, *FANCO*, *FANCP*, *FANCQ*, *FANCR*, *FANCS*, *FANCT*, *FANCU*, *FANCV*, *FANCW* [15]	Breast, ovarian, colorectal, lung, thyroid, head and neck, brain, bladder, kidney, vulval, vaginal, cervical, prostate, testicular, esophageal, gastric, pancreatic, liver, anal cancer; neuroblastoma, squamous cell carcinoma, basal cell carcinoma, melanoma, soft-tissue sarcoma, osteosarcoma [10,16,17,18,19,20,21,22].
Dyskeratosis Congenita	*DKC1*, *TERC*, *TERT*, *TINF2*, *NAF1*, *NHP2*, *NOP10*, *PARN*, *POT1*, *RTEL1*, *STN1*, *CTC1*, *WRAP53*, *POLA1*, *ACD*, *USB1*, *ZCCHC8*, *NPM1*, *MDM4*, *RPA1*, *DCLRE1B*, *TYMS-ENOSF1* [23,24]	Head and neck, colorectal, thyroid, liver, lung, esophageal, gastric, anal, pancreatic, cervical cancer; squamous cell carcinoma, basal cell carcinoma [16,17,25].
Diamond–Blackfan Anemia Syndrome	*RPS19*, *RPL5*, *RPS26*, *RPL11*, *RPL35a*, *RPS7*, *RPL8*, *RPL35*, *RPS10*, *RPS24*, *RPS17*, *RPL15*, *RPS20*, *RPS28*, *RPS29*, *RPS15a*, *RPS27*, *RPL9*, *RPL17*, *RPL18*, *RPL26*, *RPL27*, *RPL31.* *TSR2*, *GATA1*, *HEATR3 (non-RP genes)* [26]	Colorectal cancer, osteosarcoma; liver, breast, lung, thyroid, kidney, testicular, uterine, cervical, vaginal, gastroesophageal, esophageal, gastric cancer; melanoma, squamous cell carcinoma, basal cell carcinoma, rhabdomyosarcoma, soft-tissue sarcoma [16,17,27,28,29].
Shwachman–Diamond Syndrome	*SBDS*, *EFL1*, *DNAJC21*, *SRP54* [30]	Ovarian, breast, pancreatic cancer; dermatofibrosarcoma, esophageal squamous cell carcinoma, peritoneal carcinoma [17,31,32,33,34].

## Data Availability

No new data were created or analyzed in this study.

## Abbreviations

The following abbreviations are used in this manuscript:

AML	Acute myeloid leukemia
CRC	Colorectal cancer
DC	Dyskeratosis congenita
DDR	DNA damage repair
DBAS	Diamond–Blackfan anemia syndrome
FA	Fanconi anemia
HR	Homologous recombination
HSCT	Hematopoietic stem cell transplantation
IBMFS	Inherited bone marrow failure syndrome
ICL	Interstrand crosslink
MDS	Myelodysplastic syndrome
ROS	Reactive oxygen species
RP	Ribosomal protein
SDS	Shwachman–Diamond syndrome
